# *β*-Klotho inhibited the epithelial-mesenchymal transition of liver sinusoidal endothelial cells to alleviate schistosomiasis liver fibrosis

**DOI:** 10.1371/journal.ppat.1014148

**Published:** 2026-05-19

**Authors:** Tingting Jiang, Qiang Li, Zhihao Yu, Xuanyin Cui, Xiaojin Mo, Quan Chen, Yongruo Chen, Xian Li, Mengyan Wei, Zhaoyu Guo, Yuan Hu, Shizhu Li

**Affiliations:** 1 National Institute of Parasitic Diseases, Chinese Center for Disease Control and Prevention, Chinese Center for Tropical Diseases Research, National Key Laboratory of Intelligent Tracking and Forecasting for Infectious Diseases, Shanghai, China‌‌; 2 Key Laboratory of Parasite and Vector Biology, National Health Commission, WHO Centre for Tropical Diseases, Shanghai, China; 3 National Center for International Research on Tropical Diseases, Ministry of Science and Technology, Shanghai, China; 4 Big Data Institute, Nuffield Department of Population Health, University of Oxford, Oxford, United Kingdom; Rush University Medical Center, UNITED STATES OF AMERICA

## Abstract

Schistosomiasis is a neglected zoonotic disease, and the liver fibrosis induced by *Schistosoma japonicum* infection poses a significant threat to human health. Traditionally, liver fibrosis in schistosomiasis has been attributed to eggs deposited in the liver, which trigger hepatic inflammation and fibrosis. However, our study reveals that schistosomula migration to the liver induces epithelial-to-mesenchymal transition (EMT) in liver sinusoidal endothelial cell (LSEC), thereby contributing to the progression of liver fibrosis. In the early stage of *S. japonicum* infection, mice exhibited a reduction in the proportion of LSEC and impairment of their function. RNA-sequencing revealed significant alterations in β-Klotho (KLB) expression in injured LSEC. Although KLB is known to exert anti-inflammatory functions as a co-receptor for fibroblast growth factor (FGF) in the liver, its role in LSEC EMT and schistosomiasis-induced liver fibrosis was unclear. Using SK-Hep1 cells, we found that KLB knockdown exacerbated EMT, whereas KLB over-expression attenuated EMT and decreased TGFβ1 secretion from LSEC, thereby suppressing LX-2 activation. In mice infected with *S.* *japonicum*, treatment with recombinant KLB protein or AAV8-KLB increased the LSEC population, mitigated EMT in both LSEC and liver tissues, ameliorated hepatic fibrosis, and inhibited TGFβ1 pathway activation. Our study reveals that KLB suppresses LSEC EMT induced by liver-stage schistosomula and TGFβ1 secretion, thereby inhibiting HSC activation and reducing liver fibrosis. Our findings highlight KLB as a promising therapeutic target for hepatic fibrosis in schistosomiasis.

## Author summary

Schistosomiasis is a serious zoonotic parasitic disease. The deposition of *S. japonicum* eggs in the liver induces the formation of egg granulomas and promotes liver fibrosis. It is crucial to investigate the mechanisms and screen targets to inhibit its progression in schistosomiasis liver fibrosis. LSEC serve as the first line of defense against liver injury and are the first to be damaged after infection. We observed a significant increase in KLB expression in LSEC on day 12 post-infection, followed by a sustained decline. This may be associated with the EMT of LSEC induced by the infection. We demonstrated that elevating KLB expression alleviated LSEC EMT and liver fibrosis induced by *S. japonicum* infection. Further mechanistic studies revealed that KLB reduced TGFβ1 production in LSEC and suppressed TGFβR2 expression in HSC, thereby attenuating liver fibrosis. These findings identify KLB as a potential therapeutic target for treating schistosomiasis-associated liver fibrosis.

## Introduction

Schistosomiasis is a zoonotic parasitic disease caused by infection with *S. japonicum and poses* a significant threat to human health [1]. *S. japonicum* affects 78 countries worldwide, has caused approximately 250 million people to be infected, and leads to over 800 million people at risk of infection [2,3]. A large number of cases of advanced schistosomiasis still existed in China [4]. The deposition of eggs in the liver releases soluble egg antigen (SEA), inducing hepatic inflammatory responses and granuloma formation, and promoting liver fibrosis [5]. However, the regulatory mechanisms remain unclear, and specific therapeutic drugs are currently unavailable. Therefore, it is crucial to investigate the mechanisms and identify anti-fibrotic targets to reduce the burden of schistosomiasis.

EMT is the process by which epithelial cells lose their polarity and adhesion and acquire the phenotype of mesenchymal cells in pathological conditions [6]. A large number of hepatic cells, including damaged hepatocytes, activated hepatic stellate cells, and bile duct epithelial cells, can be transformed into myofibroblasts through EMT [7]. Myofibroblasts can synthesize large amounts of extracellular matrix and promote the development of liver fibrosis [8]. Increasing evidence suggests that when EMT becomes dominant, tissues and organs progress towards fibrosis [9,10]. Therefore, inhibiting liver EMT could be an effective strategy to alleviate schistosomiasis-induced liver fibrosis.

LSEC account for approximately 70% of liver non-parenchymal cells and serve as the primary barrier between the liver and the bloodstream. During infection, LSEC is the first cell type to be damaged in the liver [11]. Injury to the LSEC can promote the inflammatory response in the liver and the progression of liver fibrosis [12,13]. Our previous studies revealed that LSEC undergo EMT at 12 days post-infection with *S. japonicum* [14]. Damaged LSEC underwent EMT and transformed into myofibroblasts that produced excessive collagen [15]. Inhibiting LSEC EMT is very crucial to maintain HSC quiescence and suppress liver fibrosis at the early stage.

Klotho acts as a co-receptor for FGF. In the liver, the predominant isoform expressed is β-Klotho (KLB), which recognizes inflammatory signals transmitted by FGF19 and FGF21. KLB functions to counteract oxidative stress and suppress intrahepatic inflammatory responses [16]. The downstream signaling pathways of KLB are complex. Studies have shown that KLB can block the TGFβ signaling pathway by competitively binding to the TGFβ receptor (TGFβR). In addition, KLB inhibits the TLRs/NF-κB, ERK1/2, and Wnt/β-catenin signaling pathways, thereby attenuating EMT in retinal pigment epithelial cells and renal proximal tubule epithelial cells [17,18,19]. Klotho plays a crucial regulatory role in protecting against fibrosis in multiple organs, including the heart and kidney. Notably, mutations in the KLB gene and reduced KLB protein expression are closely associated with the development and progression of liver fibrosis [17,18,20,21]. However, the role of KLB remains unclear in schistosomiasis liver fibrosis.

In this study, we investigated the roles of KLB in regulating EMT in LSEC and in schistosomiasis liver fibrosis *in vitro* and *in vivo*. It may identify KLB as a promising target for treating schistosomiasis-related liver fibrosis by inhibiting LSEC EMT.

## Results

### Pathological changes in the LSEC of *S. japonicum*-infected mice

**Fig 1:**
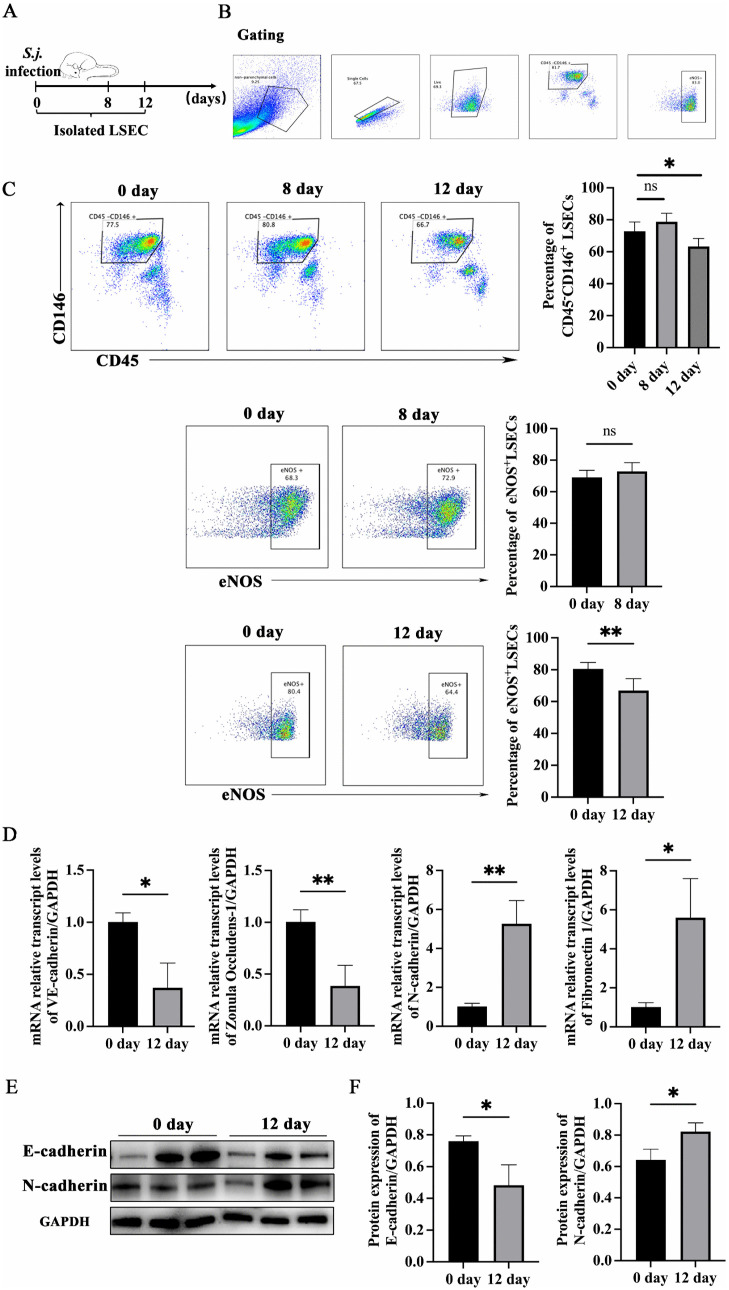
Pathological changes of LSEC in mice infected with *S. japonicum* (n = 6 mice per timepoint). **(A)** Schematic diagram of the animal experimental design. **(B)** Gating of non-parenchymal cells, single cells, and live cells. **(C)** Changes in the proportions of LSEC and eNOS^+^ LSEC on day 8 and 12 post-infection. **(D)** mRNA expression level of EMT-related marker in LSEC at 12 days after infection. **(E – F)** Protein expression level of EMT-related marker in LSEC at 12 days after infection. * P < 0.05, **P < 0.01.

The gating strategy of LSEC was shown in Fig 1B. There was no difference in the percentage of CD45^-^CD146^+^ LSEC and eNOS^+^ LSEC among liver non-parenchymal cells between the infected mice for eight days and the uninfected mice. The percentages of CD45^-^CD146^+^ LSEC and eNOS^+^ LSEC decreased significantly at 12 days post-infection (Fig 1C), indicating that LSEC were damaged at this time point.

LSEC was isolated from uninfected and 12 days post-infected mice. The mRNA levels of *VE-cadherin* and *Zonula Occludens-1*(*ZO1*) down-regulated significantly, while *N-cadherin* and *Fibronectin-1* mRNA levels upregulated after infection (Fig 1D). The protein levels of E-cadherin down-regulated, and N-cadherin upregulated in LSEC in the infected mice compared to uninfected mice (Fig 1E and 1F). These results indicated that LSEC began to undergo EMT at 12 days post-infection. These results demonstrated that *S. japonicum* infection promoted EMT in LSEC, and EMT occurred at the early stage of infection.‌‌

### KLB expression in the LSEC and the liver of the mouse after infection

**Fig 2:**
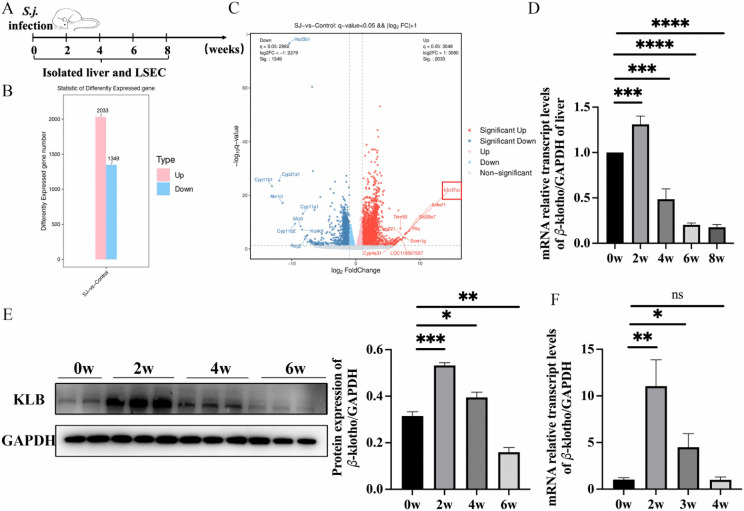
The expression of KLB in the liver and LSEC of the mouse after infection (n = 6 mice per timepoint). **(A)** Schematic diagram of the animal experimental design. **(B)** The number of differentially expressed genes in LSEC before and 12 days after *S. japonicum* infection. **(C)** The volcano plots of differentially expressed genes in LSEC before and 12 days after *S. japonicum* infection. **(D)** Expression of the *KLB* gene in the livers of mice at different time points post-infection. **(E)** The expression of KLB protein in LSEC at different time points post-infection. **(F)** The expression of the *KLB* gene in LSEC at different time points post-infection. *P < 0.05, **P < 0.01, ***P < 0.001, ****P < 0.0001.

The expression of 2033 genes was upregulated, and 1349 genes were down-regulated in LSEC after being infected with *S. japonicum* for 12 days (Fig 2B). Among them, *Klotho* increased the most significantly (Fig 2C). The *KLB* gene expression in the liver tissues increased significantly at two weeks and decreased at four weeks post-infection (Fig 2D). The *KLB* gene expression in LSEC increased at two weeks and decreased at three weeks post-infection (Fig 2F). The trend of KLB protein expression is consistent with that of *KLB* gene expression. KLB protein expression in LSEC increased at two weeks and decreased at four and six weeks after infection (Fig 2E). These results showed that KLB expression increased initially and then decreased gradually after infection.

### KLB inhibited the EMT of LSEC

**Fig 3:**
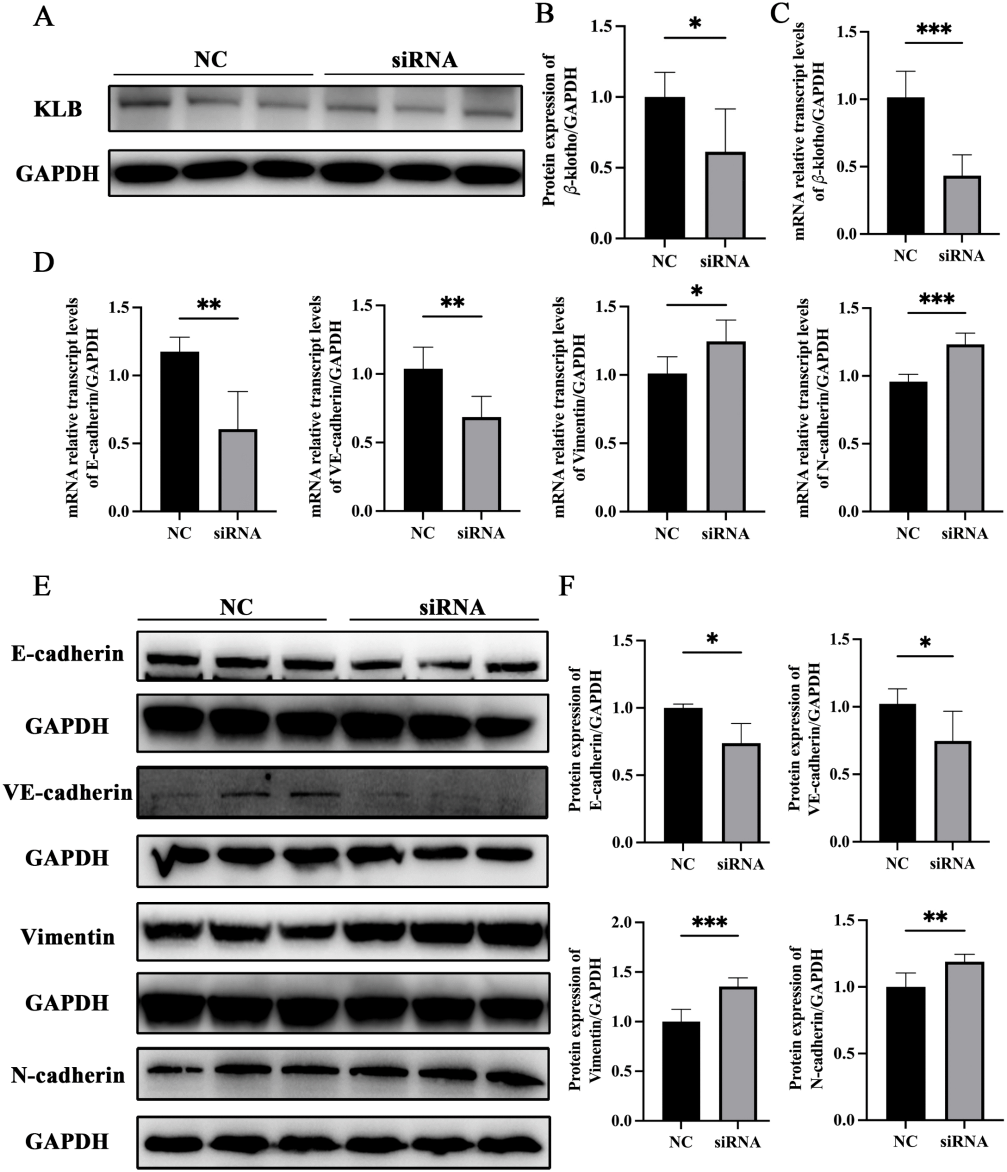
Knocking down KLB promoted EMT in SK-Hep1. **(A – B)** Expression changes and statistical graph of KLB protein after transfected with *KLB* siRNA for 48h. **(C)** Expression of the *KLB* gene after transfection with *KLB* siRNA for 48h. **(D)** The expression of EMT-related genes after transfected with *KLB* siRNA for 48h. **(E – F)** Expression changes and statistical graph of EMT-related proteins after transfected with *KLB* siRNA for 48h. *P < 0.05, **P < 0.01, ***P < 0.001.

KLB expression in SK-Hep1 (LSEC cell line) was knocked down by transfecting with *KLB* siRNA. After SK-Hep1 was transfected with *KLB* siRNA for 48 hours, the *KLB* gene and protein expressions in SK-Hep1 decreased significantly (Fig 3A–C). After knocking down the *KLB*, E-cadherin, and VE-cadherin expressions in SK-Hep1 decreased, while N-cadherin and Vimentin expressions increased (Fig 3D–F). It suggested that KLB could inhibit EMT in SK-Hep1.

**Fig 4:**
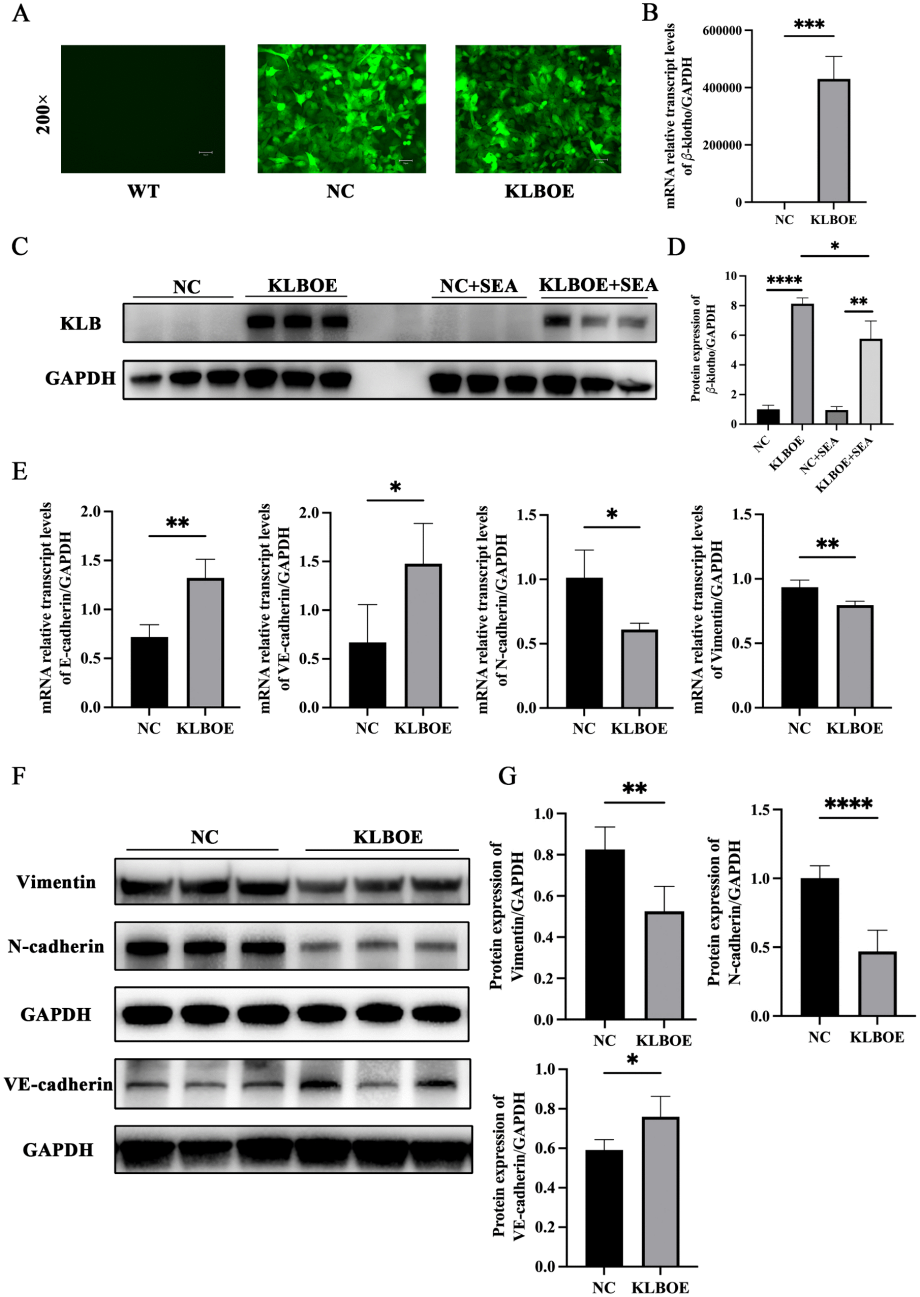
KLB overexpression attenuated EMT of LSEC. **(A)** Fluorescence graph of SK-Hep1 transfected with or without lentivirus. **(B)** The expression of the *KLB* gene in SK-Hep1 cells transfected with lentiviruses packaged with the *KLB* plasmid and the NC plasmid. **(C – D)** The expression of KLB protein in SK-Hep1 cells was induced by transfection with a lentivirus packaged with the *KLB* and NC plasmid. Moreover, SEA stimulation for 48h decreased the expression of KLB. **(E)** The expression changes of EMT-related genes transfected with lentivirus packaged with the *KLB* and NC plasmid. **(F – G)** The expression changes and statistical graph of EMT-related proteins transfected with lentivirus packaged with the *KLB* and NC plasmid. *P < 0.05, **P < 0.01, ***P < 0.001, ****P < 0.0001.

SK-Hep1 cells were transfected with lentivirus packaged with the KLB plasmid as a KLB overexpressed (KLB OE) SK-Hep1 and blank plasmid as a normal control (NC) SK-Hep1. Compared to the WT SK-Hep1 cells without virus transfection, NC SK-Hep1 and KLB OE SK-Hep1 showed significant fluorescence (Fig 4A). This indicated the lentivirus transfection was successful. The expressions of the KLB gene and protein in the KLB OE SK-Hep1 were significantly higher than those in the NC SK-Hep1 (Fig 4B–D). Moreover, after KLB OE SK-Hep1 was stimulated with SEA (80𝜇g/mL) for 48 hours, the expression of KLB protein in SK-Hep1 cells decreased significantly (Fig 4C and 4D). Compared to NC SK-hep1 cells, E-cadherin and VE-cadherin expressions in KLB OE SK-Hep1 cells increased, while Vimentin and N-cadherin expressions decreased (Fig 4E–G). These results demonstrated that KLB could inhibit EMT of SK-Hep1 cells.

### KLB inhibited EMT of LSEC induced by *S. japonicum* infection

**Fig 5:**
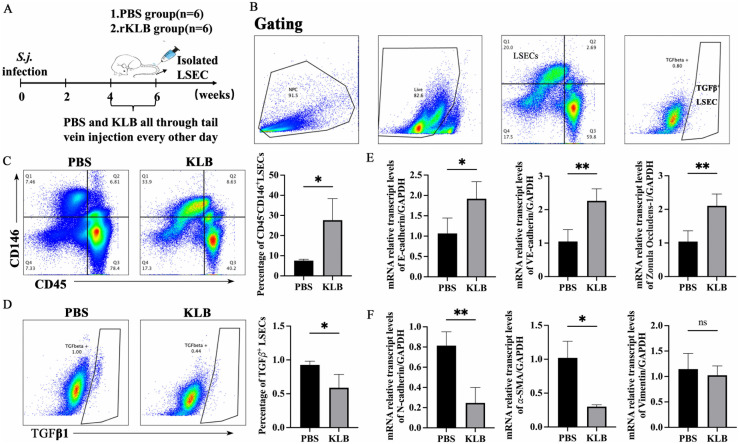
KLB attenuated EMT of LSEC induced by *S. japonicum* infection (n = 6 mice per timepoint). **(A)** Schematic diagram of the animal experimental design. **(B)** Circle gating strategy of CD45^-^CD146^+^LSEC and TGF𝛽1^+^LSEC from hepatic non-parenchymal cells. **(C)** Changes in LSEC proportion after administration of recombinant KLB protein (rKLB). **(D)** Changes in the TGFβ1^+^ LSEC proportion after rKLB administration. **(E)** The mRNA expression changes of epithelial markers *(E-cadherin*, *VE-cadherin*, *ZO1*) in LSEC after administration of rKLB. **(F)** The mRNA expression changes of mesenchymal markers (*N-cadherin*, *α-SMA*, *Vimentin*) in LSEC after administrated with rKLB. *P < 0.05, **P < 0.01.

Administration of recombinant KLB protein (rKLB) significantly increased LSEC proportion and decreased TGFβ1^+^ LSEC proportion at six weeks post-infection (Fig 5C and 5D). After administration with rKLB, the mRNA levels of epithelial markers (E-cadherin, VE-cadherin, and ZO1) upregulated significantly, while mesenchymal markers (N-cadherin and 𝛼-SMA) in primary LSEC down-regulated (Fig 5E and 5F). These results suggested that rKLB could alleviate EMT in LSEC induced by *S. japonicum* infection.

### KLB attenuated EMT and fibrosis of the liver in mice infected with *S. japonicum*

**Fig 6:**
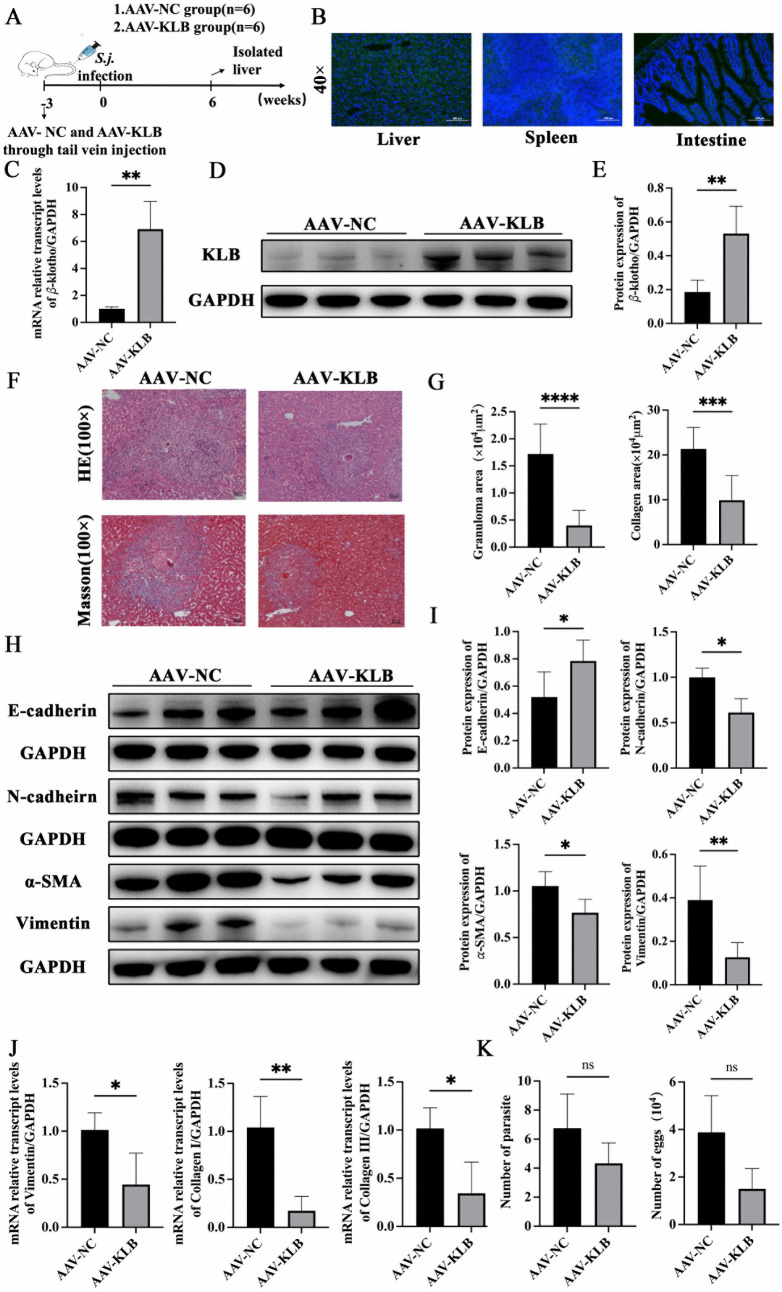
AAV8-KLB alleviated EMT and liver fibrosis in mice infected with *S. japonicum* (n = 6 mice per group). **(A)** Schematic diagram of the animal experimental design. (B) GFP fluorescence distribution in the liver, spleen, and intestinal tissues three weeks after administrated with AAV8-GFP. **(C)** The expression of *KLB* mRNA in the liver at six weeks post-infection. **(D – E)** The expression of KLB protein in the liver at six weeks post-infection. **(F)** Hematoxylin-eosin (HE) and Masson staining of the liver at six weeks post-infection. **(G)** The size of the egg granuloma and the area of collagen deposition. **(H –** **I)** The protein expressions of EMT and fibrosis-related markers. **(J)** The mRNA expressions of EMT and fibrosis-related genes. **(K)** The worm and egg burdens of infected mice in the AAV8-KLB and AAV8-NC groups. *P < 0.05, **P < 0.01, ***P < 0.001, ****P < 0.0001.

After administration with adeno-associated virus 8 (AAV8)-GFP packaged with *KLB* plasmid for three weeks, green fluorescence was strongly expressed in liver tissue, and weakly expressed in the spleen and small intestine (Fig 6B). AAV8-KLB-treated mice exhibited significantly elevated KLB expressions in liver tissues compared to AAV8-NC controls at six weeks post*-*infection (Fig 6C–E). The area of granuloma size and collagen deposition in AAV8-KLB-treated mice was smaller than that of mice in the control group (Fig 6F and 6G). The expression of E-cadherin increased, and N-cadherin, Vimentin, and α-SMA expressions decreased in the AAV8-KLB group compared to the AAV8-NC group (Fig 6H and 6I). Concurrently, hepatic mRNA expression levels of Vimentin, Collagen I, and Collagen III were significantly down-regulated (Fig 6J). No significant differences in worm and egg burdens were observed between AAV8-KLB and AAV8-NC groups (Fig 6K). After treatment with recombinant klotho protein, EMT and fibrosis levels in liver tissues were also reduced (S1 Fig). These results indicated that supplementation with KLB significantly attenuated EMT and liver fibrosis induced by *S. japonicum* infection.

### KLB inhibited TGFβ signaling pathway activation

**Fig 7:**
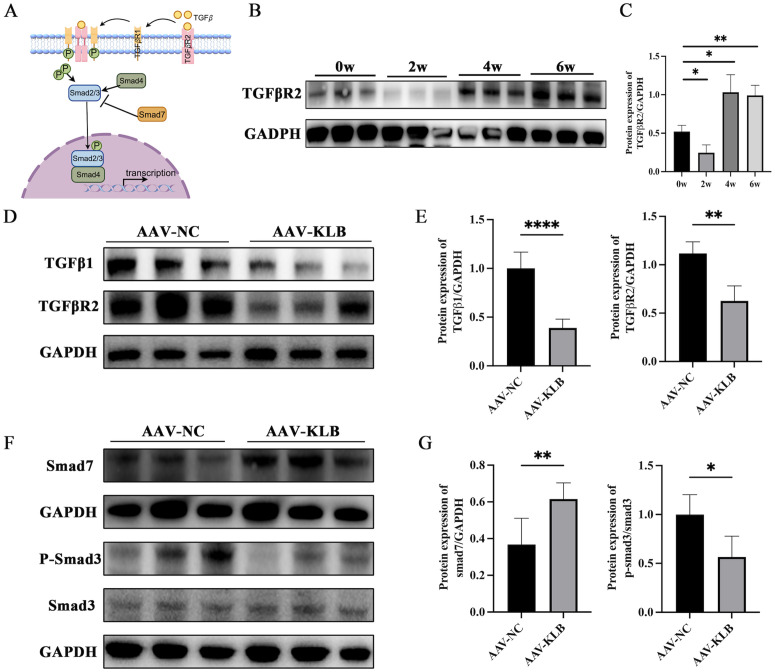
KLB suppressed the TGFβ1/Smad3 signaling pathway in liver tissues during *S. japonicum* infection (n = 6 mice per timepoint). **(A)**The canonical TGF𝛽/Smad signaling pathway. **(B – C)** Changes in TGFβR2 protein expression in the liver at different time points after infection. **(D – E)** Changes of TGFβ1 and TGFβR2 in the liver of mice in AAV8-KLB-treated and control groups at six weeks post-infection. **(F – G)** The expressions of Smad7, p-Smad3, and Smad3 in the liver of mice. *P < 0.05, **P < 0.01, ****P < 0.0001.

A schematic of the canonical TGF𝛽/Smad signaling pathway was presented in Fig 7A. TGFβR2 protein expression in the liver was down-regulated at two weeks and then elevated at four and six weeks post-infection (Fig 7B and 7C). In the AAV8-KLB-treated group, the expressions of TGFβ1, TGFβR2, and p-Smad3/Smad3 were down-regulated, while the expression of Smad7 was upregulated significantly at six weeks post-infection (Fig 7D–G). These findings indicated that KLB suppressed the TGFβ1/Smad3 signaling pathway and activated Smad7-mediated negative feedback in the liver of mice.

### KLB suppressed LX-2 activation by alleviating TGFβ secretion

**Fig 8:**
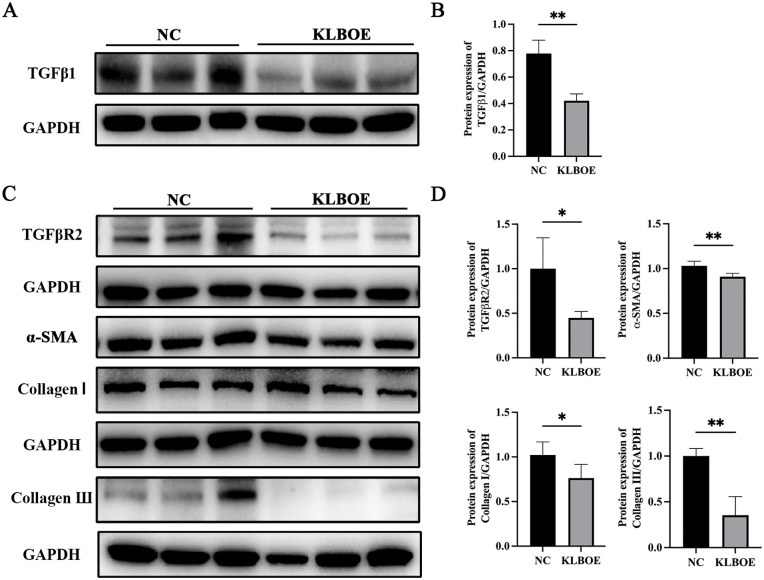
KLB suppressed LX-2 activation by inhibiting the TGFβ signaling pathway. **(A)** The protein expression of TGFβ1 in KLB OE SK-Hep1 and NC SK-Hep1 cells. **(B)** The quantitative analysis of TGFβ1 expressions. **(C)** The protein expressions of TGFβR2, α-SMA, Collagen I, and III in LX-2 cells after co-culturing with KLB OE SK-Hep1 and NC SK-Hep1 cells. **(D)** The quantitative analysis of TGFβR2, α-SMA, Collagen I, and III expressions. *P < 0.05, **P < 0.01.

KLB OE SK-Hep1 or NC SK-Hep1 cells were cultured with LX-2 cells in the chamber separately for 48 hours. SK-Hep1 cells were stimulated with 160 𝜇g/mL SEA. TGFβ1 protein expression in KLB OE SK-Hep1 was lower than that in the NC cells (Fig 8A and 8B). Collagen I, Collagen III, α-SMA, and TGFβR2 expressions in LX-2 co-cultured with KLB OE SK-Hep1 were significantly lower than those of the NC SK-Hep1 cells (Fig 8C and 8D). It suggested that KLB inhibited TGFβ1 production in LSEC and down-regulated TGFβR2 expression in HSC.

## Discussion

Schistosomiasis is a globally distributed zoonotic parasitic disease, with approximately 10% of the world’s population at risk of infection [22].*S. japonicum* is the primary species endemic to China [23]. The hepatic fibrosis induced by schistosome infection progresses to portal hypertension and cirrhosis, resulting in loss of labor capacity and imposing a severe economic burden on families and society [4,24]. In this context, the search for effective anti-fibrotic targets is of paramount importance for reducing the global burden of schistosomiasis.

Accumulating evidence has demonstrated that EMT plays a pivotal role in the progression of liver fibrosis [25]. In the liver, LSEC, HSC, and hepatocyte can undergo EMT to become myofibroblasts, promoting ECM production [26,27]. Myofibroblasts are the primary source of extracellular matrix (ECM) in fibrosis and can originate from various precursor cells. Klotho has been identified as an inhibitor of cellular EMT and exerts protective effects against fibrosis in multiple organs, including the heart and kidney [17]. Notably, mutations in the KLB gene and down regulation of KLB protein expression have been strongly implicated in the pathogenesis of liver fibrosis [21].

LSEC constitute the most abundant non-parenchymal cell population in the liver, maintaining the balance between hepatic regeneration and fibrosis [12,28]. On the one hand, the proportion of LSEC decreased and occurred EMT at 12 days post-infection. This early pathological change in LSEC may be caused by the excretory and secretory antigens released from juvenile worms during the hepatic phase. When adult worms lay eggs, the SEA released by the eggs further promotes EMT in LSEC. On the other hand, Our findings showed that the proportion of eNOS^+^ LSEC began to decrease at 12 days post-infection with *S. japonicum*. It has been reported that during acute liver injury, LSEC release nitric oxide (NO), helping maintain hepatic stellate cells (HSC) in a quiescent state [29–31]. The decline in eNOS⁺ LSEC implies that LSEC have a reduced capacity for NO production, leading to defective maintenance of HSC quiescence. Following chronic liver injury, increased reactive oxygen species and inflammatory factors in the liver cause pathological damage to LSEC, leading to loss of fenestrae and substantial TGFβ1 release, which promotes HSC activation and drives the injured liver toward fibrosis [32].

Klotho is an anti-aging gene that regulates hepatic oxidative stress, lipid metabolism, and inflammatory responses, and suppresses hepatic fibrosis [33,34]. In the liver, KLB expression exhibits a negative correlation with the level of fibrogenic gene [21]. Acetylation inactivates KLB, promoting EMT in Huh7 cells, whereas functional KLB can inhibit EMT [35]. KLB is expressed in endothelial cells, where it forms a receptor complex with FGFR to transduce signals from FGF19 and FGF21, thereby protecting cells against oxidative stress [36]. During schistosomula migration to the liver, soluble antigens released by the parasites recruit intrahepatic macrophages and neutrophils, leading to the secretion of inflammatory mediators such as IL-1β and IL-6 [37]. This induces oxidative stress in cells and triggers an intrinsic regulatory response, leading to a transient up regulation of KLB expression. As the release of soluble schistosomula and egg antigens intensifies, the intrahepatic inflammatory response becomes exacerbated, causing extensive injury to LSEC and a subsequent reduction in KLB synthesis. Notably, similar phenomena have been observed in models of liver fibrosis induced by a high-fat diet [38]. Our study revealed that KLB in LSEC was significantly upregulated in the early stage of *S. japonicum* infection. And treatment with recombinant KLB protein or AAV8-KLB could attenuate LSEC EMT and liver fibrosis. Inhibition of hepatic EMT attenuates liver fibrosis [27]. These findings indicated that KLB alleviated schistosomiasis liver fibrosis by inhibiting LSEC EMT.

After infection with *S. japonicum* for four and six weeks, the TGFβ pathway was significantly activated, as indicated by our results. The TGFβ pathway can activate HSC, promote hepatic cell transformation into fibroblasts, and induce collagen deposition [39–41]. LSEC was the main source of TGFβ in the liver [42]. Damaged LSEC could secrete TGFβ1, and active HSCs through binding to TGFβ receptors (TGFβR) on HSC [43]. This interaction triggers the activation and phosphorylation of Smad2/3, followed by their complex formation with Smad4 and subsequent nuclear translocation. The results of signaling cascades activated HSC, upregulated the secretion of pro-fibrotic cytokines, and drove hepatic fibrogenesis [44,45].

Following infection, schistosomula release soluble antigens that activate intrahepatic macrophages and neutrophils [46]. These activated cells subsequently produce pro-inflammatory cytokines such as IL-6 and IL-1β, thereby triggering intrahepatic inflammatory responses and endoplasmic reticulum oxidative stress [47]. KLB expression was significantly increased, maintaining the epithelial phenotype and stabilizing the fenestrated structure of LSEC by inhibiting EMT. The fenestrated structure of LSEC facilitated efficient exchange of materials between the liver and the bloodstream, thereby preserving intrahepatic homeostasis [32]. In addition, LSEC released soluble KLB that bound to TGFβR on the surface of HSC, blocking the TGF𝛽/Smads signaling pathway and suppressing both HSC collagen synthesis and the progression of liver fibrosis. Our study demonstrated that KLBOE SK-Hep1 (LSEC) suppressed TGFβ secretion and inhibited LX-2 cell (HSC) activation. Furthermore, KLB over expression in the liver tissue of *S. japonicum*-infected mice attenuated TGFβ1/Smad2/3 signaling activation, thereby reducing hepatic fibrosis. These findings were consistent with previous reports.

Our findings demonstrated that administration of the KLB recombinant protein exerts a therapeutic effect on liver fibrosis in schistosomiasis. Nevertheless, the practical application of the KLB protein is limited by its large molecular weight (130 kDa) [48], which complicates protein production and leads to inconsistencies across production batches. To address these challenges, future work will focus on mapping and synthesizing KLB derived epitope peptides to assess their efficacy in treating chronic liver fibrosis induced by *S.japonicum* infection.

In our study, KLB significantly inhibited EMT in LSEC and reduced TGFβ secretion, thereby maintaining LSEC epithelial phenotype. LSEC with an epithelial phenotype could maintain HSC in a resting state, thereby inhibiting liver fibrosis. These findings identify KLB as a potentially effective therapeutic target for schistosomiasis liver fibrosis.

## Materials and methods

### Ethics statement

All experiments involving C57BL/6 mice were conducted in accordance with the guidelines of the Laboratory of Animal Welfare and Ethics Committee (LAWEC) of China. The study protocol was approved by the LAWEC committee of the National Institute of Parasitic Diseases, Chinese Center for Disease Control and Prevention (Chinese Center for Tropical Diseases Research) (IPD-2023–013).

### Experimental animals and infection models

Specific-pathogen-free (SPF) female C57BL/6 mice (6–8 weeks old, body weight 20 ± 2 g) were purchased from Jihui Laboratory Animal Co., Ltd. (Shanghai, China). The mice were housed in the SPF animal facility of the National Institute of Parasitic Diseases, Chinese Center for Disease Control and Prevention. Cercariae were obtained from our institution. C57BL/6 mice were infected with *S. japonicum* cercariae (20 ± 1 cercariae/mouse) using the abdominal patch method.

### Cell isolation

C57BL/6 mice were euthanized and then sterilized by immersing them in 75% ethanol. The liver was perfused to remove red blood cells. The liver was then excised, minced, and digested at 37°C with continuous shaking for 30 minutes. The tissue homogenate was filtered to remove debris, and the resulting cell suspension was centrifuged at low speed for 5 minutes to collect the supernatant. After centrifugation, the pellet was re-suspended in Percoll solution and then centrifuged for an additional 25 minutes. The intermediate layer was carefully collected, and cells were centrifuged at 4°C to primarily obtain LSEC and Kupffer cells. Finally, LSEC were positively selected from the cell pellet using the LSEC Cell Isolation Kit (130-092-007, Miltenyi Biotec, DE).

### Flow Cytometry

The cell concentration of liver non-parenchymal cells was adjusted to 1 × 10^6^ /mL in staining buffer (2% BSA). Antibodies were used, including Fixable Viability Stain 575V (565694, BD Pharmingen, USA), CD45-PerCP-Cy5.5 (550994, BD Pharmingen, USA), CD146-AF488 (562229, BD Pharmingen, USA), eNOS-Alexa Fluor 647 (560102, BD phos flow, USA), and TGFβ1-BV421 (565638, BD Horizon, USA). LSEC were defined as CD45^-^CD146^+^ cells. They were stained with antibodies for 30 minutes at room temperature (24–26 °C) in the dark, and then washed with staining buffer. Using the transcription factor buffer set (562574, BD Pharmingen, USA), intracellular stainings for eNOS and TGFβ in LSEC were performed at 4°C in the dark. All experiments were performed using a BD FACS Verse flow cytometer (BD Biosciences, USA). For each sample, 10,000 events were acquired within the live cell gate. The gating strategy was as follows: single cells were then selected by combining FSC-H versus FSC-A gating. Live cells were identified based on low permeability to the viability dye. Subsequently, LSEC were gated as CD45-negative and CD146-positive cells. Finally, eNOS⁺ LSEC were defined as eNOS-positive cells within the LSEC population. Data were analyzed using FlowJo 10 software (TreeStar Inc., USA).

### RNA sequencing of LSEC

Primary LSEC were isolated from the liver of infected (12 days post-infection) and uninfected mice. Total RNA was extracted from LSEC using Trizol reagent, followed by removing genomic DNA contamination with DNase I. Eukaryotic mRNA was then enriched using oligo (dT) magnetic beads. The purified mRNA was fragmented into short segments and reverse transcribed into single-stranded cDNA using random hexamer primers. After purification, the double-stranded cDNA was amplified by PCR, and the resulting library was sequenced on an Illumina sequencing platform [49]. The raw sequence data reported in this paper have been deposited in the Genome Sequence Archive (https://ngdc.cncb.ac.cn/gsa/search?searchTerm = CRA038803) in the National Genomics Data Center (Nucleic Acids Res 2025).

### Animal Experiments

Animal experiments were designed as follows: ① Mice were anesthetized and sacrificed on day zero, eight, and twelve post-infection to detect changes in the proportion of LSEC and in eNOS expression. ② KLB expression in the liver tissues and LSEC was detected at zero, two, three, four, six, and eight weeks post-infection. ③ 12 mice were administered 200𝜇L PBS or 0.8 𝜇g recombinant KLB protein in 200𝜇L PBS via tail vein injection at four weeks post-infection. Another 12 mice were injected via the tail vein with 2 × 10^11^ vg of AAV8-NC or AAV8-KLB three weeks before infection. All mice were euthanized under anesthesia at six weeks post-infection. All experiments were repeated three times independently, and six mice were used per time point. Mice were infected with *S. japonicum* through their abdominal skin.

### KLB knockdown in Sk-Hep1 cells with RNA interference

SK-Hep1 cells (human LSEC line) at a concentration of 2 × 10^5^ cells/mL were seeded in 24-well plates and cultured at 37°C for 24 hours until reaching 40–50% confluence. To prepare the transfection complex, 1.25 𝜇L of 20 𝜇M siRNA was added to 30 𝜇L of 1 × riboFECT CP (C10511-05, RiboBio, China) buffer, and the mixture was gently mixed. This was followed by the addition of 3 𝜇L of riboFECT CP reagent, with gentle pipetting. After incubating at room temperature for 15 minutes, the transfection complex was added to the plate and gently mixed. Cells were then cultured at 37°C for 48 hours.

### Lentiviral-mediated KLB overexpression in SK-Hep1 cells

SK-Hep1 cells were adjusted to a concentration of 2 × 10 ^5^ cells/mL and 30% confluence. The cells were divided into three groups: a wild-type (WT) group (un-transfected control), a negative control (NC) group (transfected with an empty vector), and a KLB OE group (transfected with a KLB- over-expressing lentivirus). For the NC and KLB OE groups, equal amounts of lentivirus were added; the WT group received only complete medium. Following sixteen hours of culture, the medium was replaced with fresh complete medium. Approximately 72 hours post-infection, transfection efficiency was assessed. When the fluorescence intensity reached 80% and the cell density was 80–90%, an appropriate concentration of puromycin was added to select for stably transfected cells.

### LSEC and HSC co-culture

The eggs of *S. japonicum* were frozen and thawed repeatedly at -80°C, then ultrasonicated. The homogenate was centrifuged at 10,000 × g for 30 minutes at 4°C, and the supernatant was collected. The supernatant was then sterilized by filtration through a 0.22 𝜇m filter, and the protein concentration was determined using the BCA method. The prepared SEA was stored at -80°C for later use.

To investigate whether KLB supplementation could inhibit HSC activation under such conditions, a transwell insert with a 0.4 𝜇m polycarbonate membrane pore size for 12 well plate was used for the coculture experiments. LX-2 (human hepatic stellate cell line) was seeded into the lower chamber of 12-well plates at a density of 5 × 10^4^ cells/well. SK-Hep1 cells were transfected with NC or KLB OE vectors, and cells were seeded into the upper inserts at a density of 2 × 10^4^ cells/well (n = 6 wells per group). Complete culture medium was added to both chambers, and the cells were cultured at 37°C in a 5% CO₂ atmosphere for 24 hours. Subsequently, the medium was removed, and the upper inserts were replenished with complete medium containing 160 𝜇g/mL SEA, while the lower chambers received fresh complete medium. After 48 hours of co-culture, cells from both the upper and lower chambers were collected separately for subsequent analyses. The experiment was performed in triplicate.

### Reverse transcription-quantitative PCR

Total RNA was extracted from liver tissues and cells using Trizol reagent, and reverse transcribed into cDNA with HyperScript III RT SuperMix for qPCR with gDNA Remover (R202-02, EnzyArtisan Biotech, China). Quantitative real-time PCR was performed to measure the expression levels of target genes, including hepatic fibrosis markers (*α-SMA*, *Collagen I*, *Collagen III*, *Collagen IV*, *Fibronectin 1*), EMT markers (*E-cadherin*, *VE-cadherin*, *N-cadherin*, *Vimentin, ZO-1*), and the *KLB* gene. All primer sequences are listed in S1 and S2 Tables.

### Western blotting

Liver tissues or cells were homogenized in RIPA lysis buffer (PC101, Epizyme Biotech, China) and incubated at 4°C for 30 minutes to achieve complete cell lysis. The lysates were then centrifuged at 12,000 × g for 10 minutes, and the protein-containing supernatant was carefully collected. Protein concentration was determined using the BCA assay (PC0020, Solarbio, China), and samples were diluted to a final concentration of 2 mg/mL. The protein samples were denatured by boiling at 100°C for 10 minutes. After denaturation, samples were loaded onto SDS-PAGE gels for electrophoresis separation. Then, proteins were transferred to PVDF membranes using the EBLOT L1 fast wet transfer system according to the manufacturer’s protocol (Genscript Biotech, China). The PVDF membranes were blocked for 30 minutes and washed with 1 × TBST buffer. Primary antibodies were applied and incubated overnight at 4°C with shaking gently. The PVDF membranes were incubated with appropriate HRP-conjugated secondary antibodies at room temperature for one hour. The protein bands were visualized using enhanced chemiluminescence (ECL) substrate and imaged using a chemiluminescence detection system (Bio-Rad).

Primary antibodies included anti-GAPDH (5174S, Cell Signaling Technology, USA), anti-α-SMA (19245s, CST, USA), anti-Collagen I (bs-10423R, Bioss, China), anti-Collagen III (ab184993, Abcam, UK), anti-E-cadherin (3195s, CST, USA), anti-VE-cadherin (D87F2, Abcam, USA), anti-N-cadherin (13116s, CST, USA) anti-Vimentin (5741s, CST, USA), anti-Smad3/anti-pSmad3 (C67H9/C35A9, CST, USA), anti-Smad7 (ab216428, Abcam, UK), anti-mouse KLB (AF2619, R&D Systems, UK), anti-human KLB (ab106794, Abcam, UK), anti-TGF𝛽1 (21898–1-AP, Proteintech, China), anti-TGF𝛽R2 (ab259360, Abcam, UK), anti-Flag (F1804, Merck, DE).

### Histological analysis

After perfusion with 4% paraformaldehyde (PFA), the right liver lobe was excised and immersion-fixed in PFA. After dehydration, the tissues were embedded in paraffin, sectioned at 4 𝜇m, and dried at 60°C. The sections were prepared for HE and Masson trichrome staining. For each sample, 10–15 single-egg granulomas were selected. Using ImageJ software, the area of blue-stained inflammatory cell aggregates surrounding each egg was measured to determine the single-egg granuloma area. Additionally, the area of blue-stained collagen fibers around every single egg was quantified to evaluate the extent of hepatic fibrosis. The mean values from all measured granulomas were calculated for each sample.

### Statistical analysis

All data were analyzed using GraphPad Prism 9.0.0 software. Quantitative variables are presented as mean ± standard deviation. Intergroup comparisons were performed using Student’s t-test, with p < 0.05 considered statistically significant.

## Supporting information

S1 Data An Excel spreadsheet containing the raw data for all figures. (XLSX)

S1 Table Primer sequences for mouse reverse transcription-quantitative PCR. (DOCX)

S2 Table Primer sequences for human reverse transcription-quantitative PCR. (DOCX)

S1 Fig Klotho recombinant protein alleviated liver EMT and fibrosis in *S.* *japonicum*-infected mice. (A) The mRNA expressions of fibrosis-associated genes (*𝛼-SMA*, *Collagen I*, *Collagen III*, *Collagen IV*) in murine liver tissues. (B) The protein expressions of markers related to EMT and liver fibrosis (E-cadherin, 𝛼-SMA, Collagen I). (C) Statistical analysis of protein expressions related to EMT and liver fibrosis. *P < 0.05, **P < 0.01.
(TIF)

S2 Fig *S. japonicum* SSA and SEA both induced EMT in Sk-Hep1(human LSEC line). (A) Soluble schistosomulum antigen (SSA) stimulated the expression of EMT-related proteins in SK-Hep1. (B) The quantitative analysis of E-cadherin and N-cadherin expressions after SSA stimulation. (C) SEA stimulated the expression of EMT-related proteins in SK-Hep1. (D) The quantitative analysis of E-cadherin and N-cadherin expressions after SEA stimulation. *P < 0.05, **P < 0.01.
(TIF)

S3 Fig Graphical abstract. (TIF)
