# Supplementary material for: β-Klotho inhibited the epithelial-mesenchymal transition of liver sinusoidal endothelial cells to alleviate schistosomiasis liver fibrosis
Source: PLoS Pathog. 2026 May 19;22(5):e1014148. doi: 10.1371/journal.ppat.1014148 (PMC13186334; doi:10.1371/journal.ppat.1014148)
Supplement: S1 Table — (DOCX) [file ppat.1014148.s002.docx]

**Supplementary Table S1**

**Primer sequences for mouse reverse transcription-quantitative PCR.**

| Gene | 5’-3’ |
| --- | --- |
| GAPDH | F: CATCACTGCCACCCAGAAGACTG  R: ATGCCATGAGCTTCCCGTTCAG |
| E-cadherin | F: GGTCATCAGTGTGCTCACCTCT  R: GCTGTTGTGCTCAAGCCTTCAC |
| VE-cadherin | F: GAACGAGGACAGCAACTTCACC  R: GTTAGCGTGCTGGTTCCAGTCA |
| N-cadherin | F: CCTCCAGAGTTTACTGCCATGAC  R: CCACCACTGATTCTGTATGCCG |
| Zonula Occludens-1 | F：GTTGGTACGGTGCCCTGAAAGA  R：GCTGACAGGTAGGACAGACGAT |
| Vimentin | F: CGGAAAGTGGAATCCTTGCAGG  R: AGCAGTGAGGTCAGGCTTGGAA |
| α-SMA | F: CTGGTATTGTGCTGGACTCTG  R: GATCTTCATGAGGTAGTCGGT |
| Collagen Ⅰ | F: CCTCAGGGTATTGCTGGACAAC  R: TTGATCCAGAAGGACCTTGTTTG |
| Collagen Ⅲ | F: GACCAAAAGGTGATGCTGGACAG  R: CAAGACCTCGTGCTCCAGTTAG |
| Collagen Ⅳ | F: ATGGCTTGCCTGGAGAGATAGG  R: TGGTTGCCCTTTGAGTCCTGGA |
| KLB | F: GAAAGAGTCCACGCCAGACATG  R: CAGGTGAGGATCGGTAAACTGC |
