# Supplementary material for: β-Klotho inhibited the epithelial-mesenchymal transition of liver sinusoidal endothelial cells to alleviate schistosomiasis liver fibrosis
Source: PLoS Pathog. 2026 May 19;22(5):e1014148. doi: 10.1371/journal.ppat.1014148 (PMC13186334; doi:10.1371/journal.ppat.1014148)
Supplement: S2 Table — (DOCX) [file ppat.1014148.s003.docx]

**Supplementary Table S2**

**Primer sequences for human reverse transcription-quantitative PCR.**

| Gene | 5’-3’ |
| --- | --- |
| GAPDH | F: AAGGTGAAGGTCGGAGTCAAC  R: GGGGTCATTGATGGCAACAATA |
| E-cadherin | F: CCCAATACATCTCCCTTCACAG  R: CCACCTCTAAGGCCATCTTTG |
| VE-cadherin | F: GAAGCCTCTGATTGGCACAGTG  R: TTTTGTGACTCGGAAGAACTGGC |
| N-cadherin | F: CCTCCAGAGTTTACTGCCATGAC  R: GTAGGATCTCCGCCACTGATTC |
| Vimentin | F: AGGCAAAGCAGGAGTCCACTGA  R: ATCTGGCGTTCCAGGGACTCAT |
| KLB | F: AAGAGTCCACGCCAGATGTGCA  R: CCACACGTACAGATGAGGATCG |
